# Microarray data and pathway analyses for human microvascular endothelial cells supplemented with low dose vitamin D or niacin during lipotoxicity

**DOI:** 10.1016/j.dib.2019.104490

**Published:** 2019-09-09

**Authors:** Kia M. Peters, Nica M. Borradaile

**Affiliations:** Department of Physiology and Pharmacology, Schulich School of Medicine and Dentistry, Western University, London, Ontario, Canada

## Abstract

Low dose niacin and vitamin D can directly improve human microvascular endothelial cell angiogenic function under lipotoxic conditions Peters et al.,2019. Despite exerting similar benefits on *in vitro* angiogenic function, these vitamins are known to signal through independent receptors, raising the possibility that differential changes in gene expression may underlie these effects. Here we provide data collected using Affymetrix GeneChip microarrays to compare gene expression in human microvascular endothelial cells treated for 16 h with growth medium containing BSA alone, or BSA complexed with the saturated fatty acid palmitate, and supplemented with 10 μM niacin or 10 nM vitamin D (1,25-dihydroxyvitamin D3). Data sets of differential gene expression included many genes involved in cellular stress responses. Pathway analyses of genes specific to vitamin D treatment identified a robust overrepresentation of pathways related to the cell cycle and DNA replication and repair.

**Table:** Specifications Table

Subject area	*Vascular biology*
More specific subject area	*Endothelial cell biology*
Type of data	*Tables, schematic images*
How data was acquired	*Affymetrix GeneChip RNA microarray, RMA and statistical analyses*
Data format	*Filtered, analyzed*
Experimental factors	*Human microvascular endothelial cells were incubated for 16 h with growth medium containing either BSA alone or BSA complexed with palmitate and supplemented with niacin or 1,25-dihydroxyvitamin D3.*
Experimental features	*RNA isolation and global gene expression analyses*
Data source location	*London, Ontario, Canada*
Data accessibility	*Data is within this article*

**Table:** 

**Value of the data** • A global gene expression analysis of human endothelial cells treated with niacin or vitamin D under lipotoxic conditions • These data may be useful for the comparison with microarray data from other cell or tissue types treated with niacin or vitamin D or treated under high fatty acid conditions • Genes and pathways identified as differentially expressed in this data set could be investigated in future studies of the angiogenic or survival mechanisms of niacin and vitamin D under high fatty acid conditions

## 1 Data

**Fig. 1:**
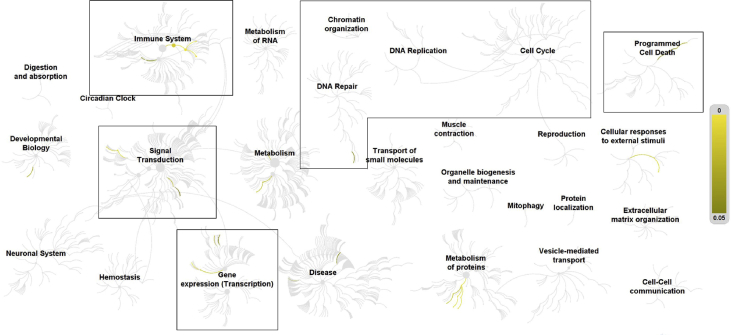
Pathways overrepresented in the list of differentially expressed genes in human microvascular endothelial cells treated with palmitate compared to BSA control. Top level pathways are represented by central nodes, with nodes in the outer rings representing sub-pathways. Relationships between nodes are represented by arcs (edges). Significantly (*p* < 0.05) overrepresented (enriched) pathways and relationships are indicated by increasing yellow signal. Black boxes in Fig. 1, Fig. 2, Fig. 3 highlight the top-level pathways and associated sub-pathways identified to have significant overrepresentation in all 3 comparisons. Reactome identifiers, pathway names, and genes identified in significantly overrepresented pathways are provided in Supplementary material.

**Fig. 2:**
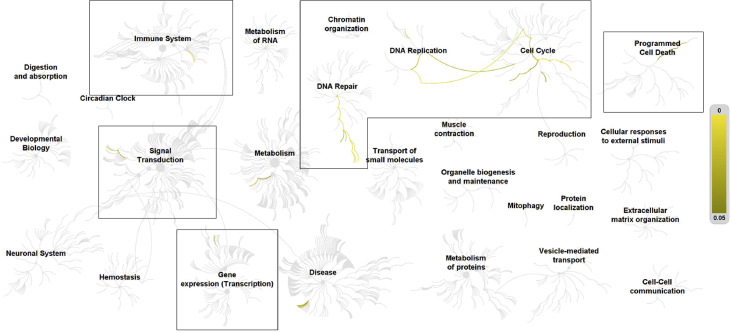
Pathways overrepresented in the list of differentially expressed genes in human microvascular endothelial cells treated with palmitate supplemented with 10 μM niacin, compared to BSA control. Top level pathways are represented by central nodes, with nodes in the outer rings representing sub-pathways. Relationships between nodes are represented by arcs (edges). Significantly (*p* < 0.05) overrepresented (enriched) pathways and relationships are indicated by increasing yellow signal. Black boxes in Fig. 1, Fig. 2, Fig. 3 highlight the top-level pathways and associated sub-pathways identified to have significant overrepresentation in all 3 comparisons. Reactome identifiers, pathway names, and genes identified in significantly overrepresented pathways are provided in Supplementary material.

**Fig. 3:**
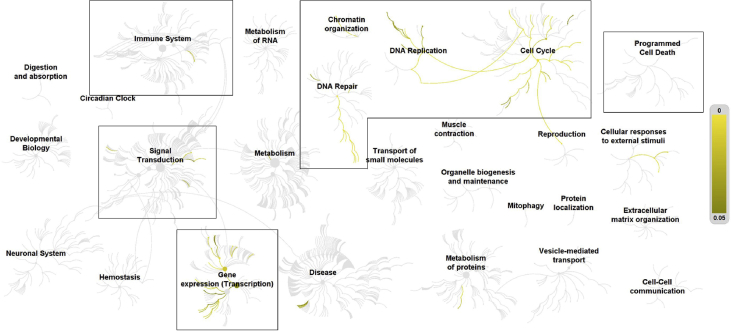
Pathways overrepresented in the list of differentially expressed genes in human microvascular endothelial cells treated with palmitate supplemented with 10 nM vitamin D, compared to BSA control. Top level pathways are represented by central nodes, with nodes in the outer rings representing sub-pathways. Relationships between nodes are represented by arcs (edges). Significantly (*p* < 0.05) overrepresented (enriched) pathways and relationships are indicated by increasing yellow signal. Black boxes in Fig. 1, Fig. 2, Fig. 3 highlight the top-level pathways and associated sub-pathways identified to have significant overrepresentation in all 3 comparisons. Reactome identifiers, pathway names, and genes identified in significantly overrepresented pathways are provided in Supplementary material.

Microarray analyses comparing mRNA isolated from human microvascular endothelial cells (HMVEC) following 16 h treatment in growth medium containing bovine serum albumin (BSA) or 0.5 mM palmitate supplemented with 10 μM niacin or 10 nM vitamin D revealed significantly (*p* < 0.05) altered expression of many cellular stress response genes. These included genes involved in ER stress, oxidative stress, and inflammation, at a fold change of greater than 2 [1] (Supplemental material). These data were consistent with subsequent Reactome pathway analyses (Fig. 1, Fig. 2, Fig. 3, and Supplemental material). Pathway analyses of differentially expressed genes specific to vitamin D treatment identified robust overrepresentation of genes involved in cell cycle regulation, DNA replication, DNA repair, and signal transduction (Fig. 2, and Supplemental material).

## 2 Experimental design, materials and methods

### 2.1 Endothelial cell culture and treatments

Primary HMVEC (Lonza, Walkerville, MD USA) were maintained in Medium 199 (Life Technologies, Carlsbad, CA, USA) supplemented with EGM-2MV SingleQuots (Lonza) and subcultured according to manufacturer's protocol. For experiments, cells from a single donor were used at subculture 5. For lipotoxic treatments, cells were exposed to media supplemented with 0.5 mM palmitic acid complexed to bovine serum albumin (BSA) at a molar ratio of 2:1. Cell monolayers at 80% confluence were treated for 16 h with media containing either BSA, palmitate, palmitate with 10 nM vitamin D (1,25-dihydroxyvitamin D3; Sigma Aldrich), solubilized in dimethyl sulfoxide (DMSO), or palmitate with 10 μM niacin, solubilized in cell culture grade water. DMSO (2 μl/ml of media) was used as the vehicle control. A total of twelve samples (three BSA, three palmitate, three palmitate plus vitamin D, and three palmitate plus niacin) were generated for subsequent gene expression analyses.

### 2.2 RNA isolation, quality assessment, probe preparation and gene chip hybridization

Following treatment, cells were lysed and RNA was isolated following a standard QIAGEN protocol as previously described. RNA concentrations and purity were measured using a NanoDrop spectrophotometer (Thermo Scientific). Samples were diluted to a concentration of 200 ng/μL in nuclease-free water and RNA was stored at −80 °C prior to microarray analyses. All sample labeling and GeneChip processing were performed at the London Regional Genomics Centre (Robarts Research Institute). RNA quality was assessed using an Agilent 2100 Bioanalyzer (Agilent Technologies) and the RNA 6000 Nano kit (Caliper Life Sciences). End labeled single stranded complementary DNA was prepared from 200 ng of total RNA and hybridized, for 16 hours at 45 °C, to Human Gene 2.1 ST arrays, as described in the Affymetrix Technical Analysis Manual. All liquid handling steps were performed using the GeneChip Fluidics Station 450 and GeneChips were scanned with the GeneChip Scanner 3000 7G (Affymetrix) controlled by Command Console v3.2.4.

### 2.3 Statistical analyses of changes in global gene expression

Microarray probe data were imported into Partek Genomics Suite v6.6 using the robust multi-array average (RMA) algorithm [2]. Partek was used to determine gene level ANOVA p-values and fold changes. Filtered gene lists were generated for expression changes of greater than 2.0-fold and having a p-value of less than 0.05 (Supplementary material).

### 2.4 Pathway analyses

Lists of genes exhibiting at least a 2.0-fold change in expression with a p-value less than 0.05 were used for gene ontology (GO) and KEGG pathway enrichment analyses (Supplementary material). Over-represented transcripts were also imported into the open-source Reactome curated pathway data base (v58) (http://www.reactome.org/) for further analyses. Pathway overview schematics, indicating significantly represented (p < 0.05) nodes (pathways) and relationships between nodes were generated for each list of differentially expressed genes.
